# Supplementary material for: High-efficiency reinforcement learning with hybrid architecture photonic integrated circuit
Source: Nat Commun. 2024 Feb 5;15:1044. doi: 10.1038/s41467-024-45305-z (PMC10844654; doi:10.1038/s41467-024-45305-z)
Supplement: Supplementary file 1 — Supplementary Information [file 41467_2024_45305_MOESM1_ESM.pdf]

Supplementary Information for

High-efficiency Reinforcement Learning with Hybrid Architecture Photonic Integrated Circuit

Xuan-Kun Li,[1,2] Jian-Xu Ma,[3] Xiang-Yu Li,[3] Jun-Jie Hu,[1,2] Chuan-Yang Ding,[1,2] Feng-Kai Han,[1,2] Xiao-Min Guo,[3] Xi Tan,[1,2] Xian-Min Jin[1,2,3,4*]

[1]Center for Integrated Quantum Information Technologies (IQIT), School of Physics and Astronomy and State Key Laboratory of Advanced Optical Communication Systems and Networks, Shanghai Jiao Tong University, Shanghai 200240, China.

[2]Hefei National Laboratory, Hefei, 230088, China

[3]TuringQ Co., Ltd., Shanghai 200240, China

[4]Chip Hub for Integrated Photonics Xplore (CHIPX), Shanghai Jiao Tong University, Wuxi 214000, China

[*]E-mail: xianmin.jin@sjtu.edu.cn

This PDF file includes:

Supplementary Sections 1 to 9

Figs.S1 to S13

Tables S1 to S3

Supplementary Section 1: Device measurement of HyArch PIC

Figure S1 demonstrates the details of PIC hardware and device performance. The photograph of photonic packaging of HyArch PIC is shown in Fig. S1(a). Inset shows top-down views of the packaged HyArch PIC. The grating coupler array is coupled with the fiber array (FA) and cured by optical glue, and the electrical pads are connected to the PCB by wire bonding. Fig. S1(b) is microscope image of the core components: grating coupler (GC), 1×2 and 2×2 multi-mode interferometer (MMI) and push-pull Mach Zehnder Interferometer (MZI) modulator. We sweep the 10 mW tunable CW laser wavelength to measure the optical response of the reference grating coupler and HyArch PIC, the measurement result is shown in Fig. S1(c). Fiber-chip coupling loss is 3.45 dB/port measured by reference GC and the minimum system end-to-end loss (U and M0) is 13.45 dB, which is measured when all M0 links are in-phase. Fig. S1(d) is the modulation range measurement results of all OCTOPUS links. The maximum modulation range difference within the same module is 0.0182, showing the good uniformity between different branches, and demonstrating the stability of foundry manufacturing. Measuring these hardware-inherent errors allows us to calibrate them in advance when performing optical calculations, enabling more accurate and highly parallel optical calculations. The transmission of the MZI in U(3) module as a function of an applied voltage shows the extinction ratio (ER) close to 30 dB. Fig. S1(e) demonstrates the phase sweep curve of MZI modulator, showing an extinction ratio (ER) close to 30 dB.

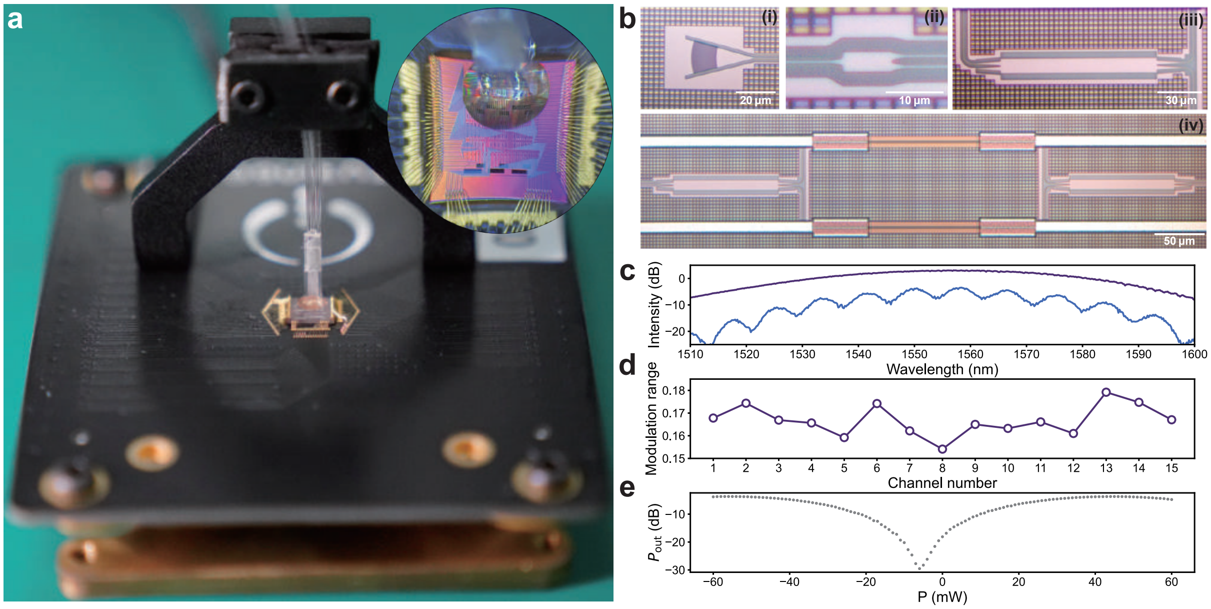

Fig. S1: **Microscope image of the HyArch PIC and performance details.** **(a)** Photograph of the photonic packaging of HyArch PIC. Insets show top-down views of the packaged HyArch PIC. The grating coupler array is coupled with the fiber array (FA) and cured by optical glue, and the electrical pads are connected to the PCB by wire bonding. **(b)** Display of key optical elements in detail. (i) grating coupler for I/O; (ii) and (iii) 1×2 and 2×2 power splitters; (iv) push-pull MZI modulator, that top and bottom arm are both phase-tunable through their independent heater. **(c)** Measured optical response of reference grating coupler (purple curve) and HyArch PIC (blue curve). **(d)** The modulation range of all 15 OCTOPUS links. **(e)** The transmission of the MZI in the U(3) module is shown as a function of the applied power, revealing an extinction ratio (ER) close to 30 dB.

Supplementary Section 2: OCTOPUS module characterization

Figure S2 shows the control loop used to perform the OCTOPUS module characterization. Due to the existence of inherent phase and nonlinear modulation effects in the PIC, we begin with the implementation of the simulated annealing (SA) algorithm. This involves continuously adjusting the configuration of each phase to maximize the output power, ensuring the coherent superposition of each link. We modified the built-in SA algorithm provided from the Python package scikit-opt [1] to ensure unidirectional optimization towards the maximum value. There are 16 individual tunable phase items within a single OCTOPUS module, and the entire optimization process involves approximately 300 iterations. Figure S3 depicts the optimization curve for all three modules. The imbalance factor k and half-wave power P_π are wavelength-dependent, necessitating an input wavelength scan for optimizing the overall joint spectrum. By performing two-dimensional scanning on $\Delta_{\mathrm{P}}^{\mathrm{A}}$ and $\Delta_{\mathrm{P}}^{\mathrm{B}}$ at different wavelengths λ, A-B joint spectra at different wavelengths $I(\Delta_{\mathrm{P}}^{\mathrm{A}}, \Delta_{\mathrm{P}}^{\mathrm{B}}, \lambda)$ can be obtained. The zero-point opening (ZPO) is calculated for these joint spectra, and the optimal wavelength λ_{opt} corresponds to the wavelength at which the ZPO is minimized.

Subsequently, unit calibration is conducted to establish the correlation between modulation power P and the output value of the MZI, denoted as $V(\Delta_{\mathrm{P}})$, as well as the phase shifter $\Phi(P)$. This process is equivalent to fitting the transfer function T for these units. In the push-pull MZI modulator structure, the input optical field E_{in} is split into two beams by a beam splitter. These beams undergo independent phase modulation with modulation powers P_1 and P_2 respectively. Then, the modulated beams are recombined using a beam combiner to generate the output optical field E_{out}. The general transmission function of push-pull MZI with splitter imbalance factor k can be expressed as:

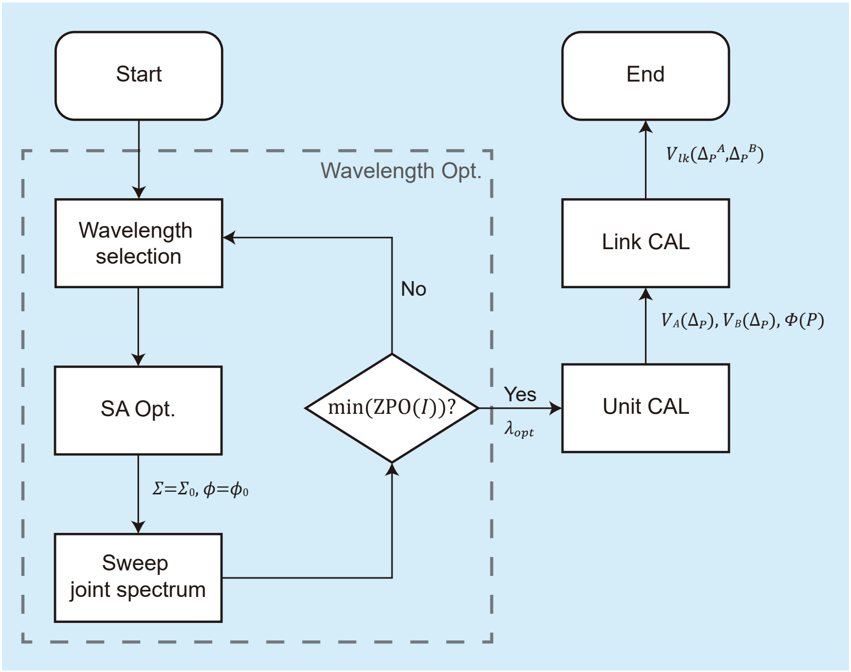

Fig. S2: **Flow chart for the OCTOPUS module characterization**

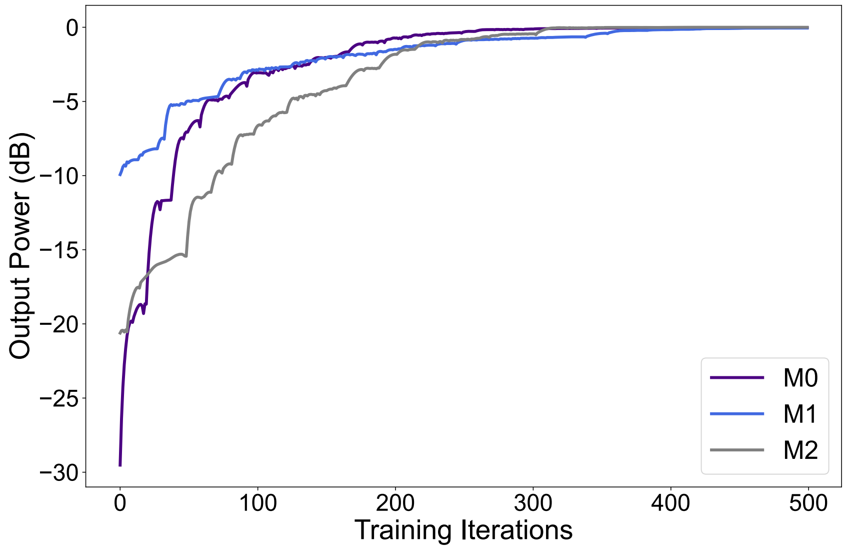

Fig. S3: **Simulated annealing optimization curve of three OCTOPUS modules.**

$$
\begin{aligned}
E_{\text{out}} &= \frac{E_{\text{in}}}{2}\left((1-k)e^{\frac{j\theta_1}{2}} + (1+k)e^{\frac{j\theta_2}{2}}\right) \\
&= \frac{E_{\text{in}}}{2}\left((1-k)e^{\frac{j(\Sigma_\theta+\Delta_\theta)}{2}} + (1+k)e^{\frac{j(\Sigma_\theta-\Delta_\theta)}{2}}\right) \\
&= E_{\text{in}}\left(\cos\left(\frac{\Delta_\theta}{2}\right) - jk\sin\left(\frac{\Delta_\theta}{2}\right)\right)e^{j\Sigma_\theta/2}
\end{aligned}
\tag{S1}
$$

where $\Delta_\theta = (\theta_1 - \theta_2)/2$ and $\Sigma_\theta = (\theta_1 + \theta_2)/2$, with θ_1 and θ_2 determined by the modulation powers P_1 and P_2 respectively. We will fit the following model:

$$
\begin{gathered}
T_{\text{PS}}(P) = C_0\left|C_1 e^{i\phi(P)} + 1 - C_1\right| \\
\phi(P) = C_3\left(P + C_2\right) \\
T_{\text{MZI}}\left(\Delta_{\text{P}}\right) = D_0\left|D_1\left(\left(0.5 + D_4\right)e^{i\frac{\Delta_\theta(\Delta_{\text{P}})}{2}} + \left(0.5 - D_4\right)e^{-i\frac{\Delta_\theta(\Delta_{\text{P}})}{2}}\right)e^{iD_5} + 1 - D_1\right| \\
\Delta_\theta\left(\Delta_{\text{P}}\right) = D_3\left(\Delta_{\text{P}} + D_2\right)
\end{gathered}
\tag{S2}
$$

where $C_i (i = 0 \sim 3)$ and $D_i (i = 0 \sim 5)$ represent fitting parameters. Each module requires unit calibration for 10 MZIs and 6 phase shifters. We denote the calibrated mapping relationship as the value-power functions $V(P)$ and $V(\Delta_{\text{P}})$, and utilize these functions for subsequent calibration and encoding.

Finally, link calibration uses the transfer function of the entire OCTOPUS link as the model for higher-order calibration, aiming to eliminate the coupling term between individual units in the link. Each link consists of two cascaded push-pull MZIs (labeled as A and B) and a tail phase shifter to compensate for the phase. Assuming identical imbalances for different push-pull MZIs, the transmission function of each link can be expressed as:

$$
\begin{aligned}
E_{\text{out}}^{lk} &= E_{\text{in}}\left(\cos\left(\frac{\Delta_\theta^{\text{A}}}{2}\right) - jk_{\text{A}}\sin\left(\frac{\Delta_\theta^{\text{A}}}{2}\right)\right)\left(\cos\left(\frac{\Delta_\theta^{\text{B}}}{2}\right) - jk_{\text{B}}\sin\left(\frac{\Delta_\theta^{\text{B}}}{2}\right)\right)e^{j\frac{\Sigma_\theta^{\text{A}}+\Sigma_\theta^{\text{B}}}{2}+\phi} \\
&= E_{\text{in}}\left(\cos\left(\frac{\Delta_\theta^{\text{A}}}{2}\right)\cos\left(\frac{\Delta_\theta^{\text{B}}}{2}\right) - jk\sin\left(\frac{\Delta_\theta^{\text{A}}+\Delta_\theta^{\text{B}}}{2}\right) - k^2\sin\left(\frac{\Delta_\theta^{\text{A}}}{2}\right)\sin\left(\frac{\Delta_\theta^{\text{B}}}{2}\right)\right)e^{j\frac{\Sigma_\theta^{\text{A}}+\Sigma_\theta^{\text{B}}}{2}+\phi}
\end{aligned}
\tag{S3}
$$

where the phase term ϕ represents the total modulated phase of the links to offset the inherent phase and achieve coherent superposition of each link. We fit the following expression using data from sweeping $I(V_{\mathrm{A}}, V_{\mathrm{B}}, \phi, \lambda_{\mathrm{opt}})$:

$$T_{lk}\left(\Delta_P^{\mathrm{A}}, \Delta_P^{\mathrm{B}}\right) = \cos\left(\frac{\Delta_\theta^{\mathrm{A}}}{2}\right)\cos\left(\frac{\Delta_\theta^{\mathrm{B}}}{2}\right) + \mathbf{S_1}\cdot\sin\left(\frac{\Delta_\theta^{\mathrm{A}}+\Delta_\theta^{\mathrm{B}}}{2}\right) + \mathbf{S_2}\cdot\sin\left(\frac{\Delta_\theta^{\mathrm{A}}}{2}\right)\sin\left(\frac{\Delta_\theta^{\mathrm{B}}}{2}\right)$$
$$\Delta_\theta = \frac{\pi}{P_\pi}\left(\Delta_P + \Delta_{P_0}\right) \tag{S4}$$

where $\mathbf{S_1}$ and $\mathbf{S_2}$ are the complex link error coefficient, requiring separate fitting for their real and imaginary parts. Following link calibration, denoted as $T_{lk}\left(\Delta_P^{\mathrm{A}}, \Delta_P^{\mathrm{B}}\right) = V'(\Delta_P^{\mathrm{A}})V'(\Delta_P^{\mathrm{B}})$, the A-B joint spectra of all 15 OCTOPUS links closely resemble the balance model, as illustrated in Fig. S4.

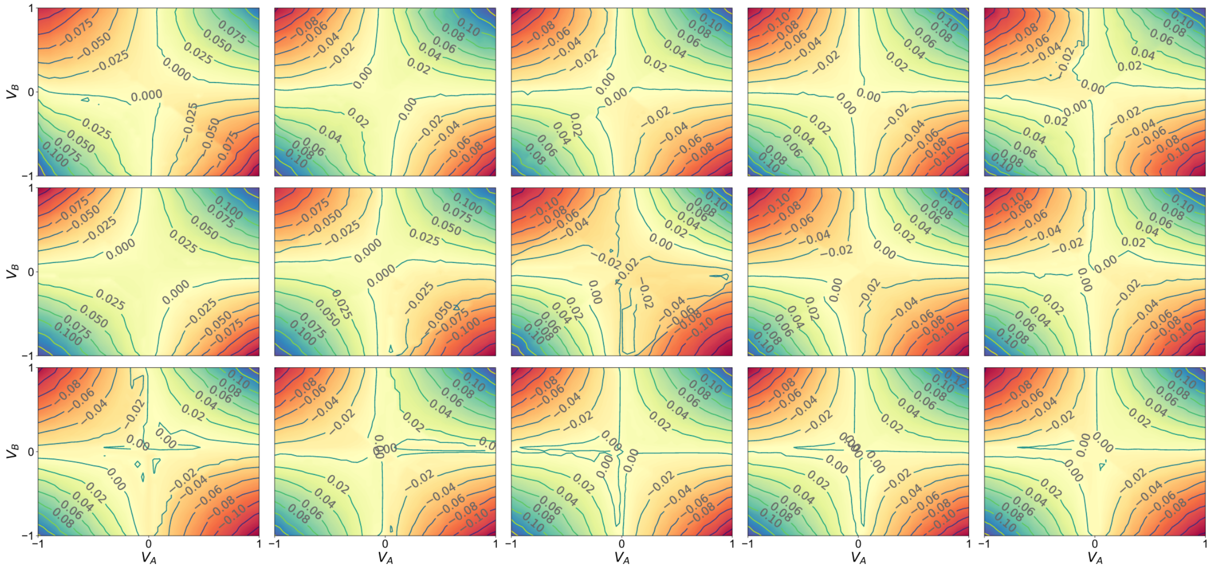

Fig. S4: **A-B joint spectra after link calibration.**

Supplementary Section 3: An overview of reinforcement learning and Q-Learning

Reinforcement Learning (RL) is a subfield of machine learning (ML) that focuses on training agents to make sequential decisions in an environment to maximize a cumulative reward signal [2]. Unlike traditional supervised learning, where the algorithm is provided with labeled examples, and unsupervised learning, where patterns are learned from unlabeled data, RL operates through interactions with an environment, learning to take actions that lead to the most favorable outcomes over time. This unique aspect sets RL apart within the machine learning landscape. In RL, an agent learns to navigate an environment by trial and error, receiving feedback in the form of rewards or penalties after each action taken. The agent's objective is to discover a policy, a strategy that maps states to actions, which optimizes its long-term expected cumulative reward. This dynamic and adaptive learning paradigm is particularly well-suited for problems where the optimal decision-making strategy is not known beforehand and needs to be learned through exploration.

Q-Learning is a fundamental algorithm within RL that played a pivotal role in its development [3]. Q-Learning is a model-free RL method, meaning it doesn't require a model of the environment to make decisions. Instead, it uses a Q-table (Quality table) to approximate the expected cumulative reward of taking a particular action in a specific state. Through iterative updates, Q-Learning refines its estimates of the Q-values, allowing the agent to learn an optimal policy over time. Q-Learning's significance in the development of RL lies in its ability to tackle complex problems with discrete action and state spaces, paving the way for the exploration of RL in a variety of domains, including robotics, game playing, and autonomous systems. Its simple yet powerful approach laid the foundation for more advanced RL techniques, such as Deep Q-Networks (DQNs) [4], which combine Q-Learning with deep neural networks to handle high-dimensional input spaces like images. In Fig. S5, we present the classification of RL

and illustrate the interrelationship among ML, RL, and Q-learning.

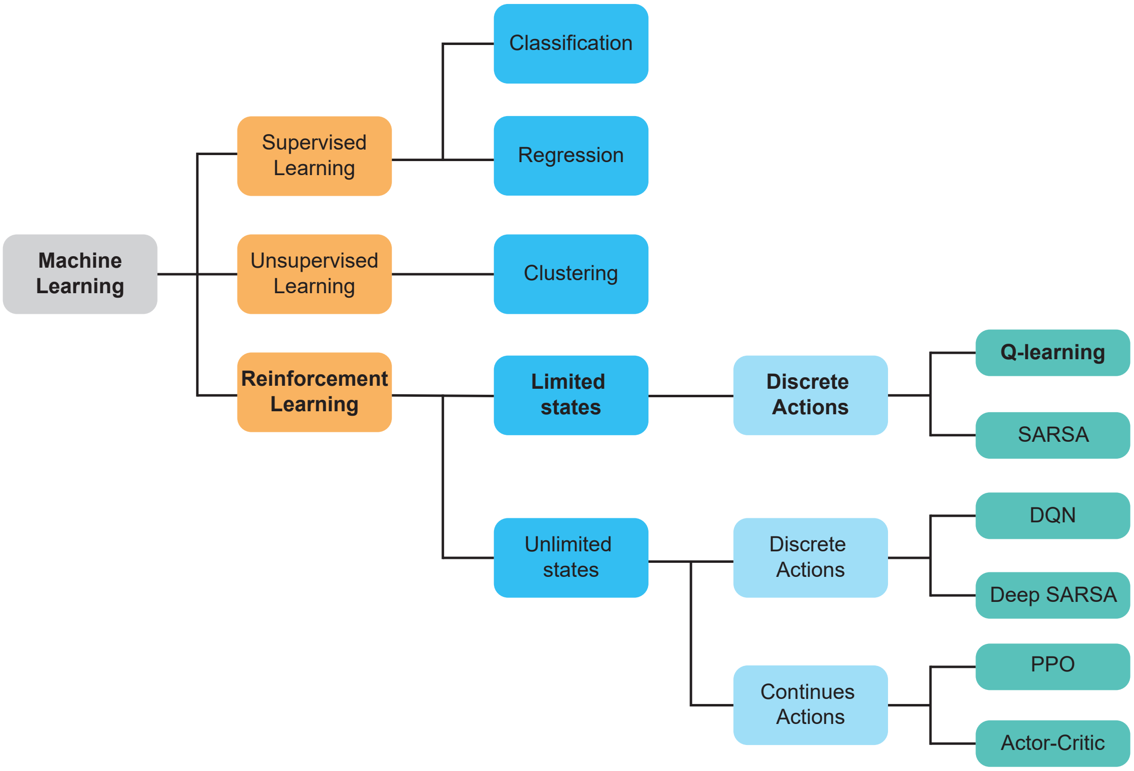

Fig. S5: **Relationship among machine learning (ML), reinforcement learning (RL), and Q-learning (QL).**

Within the context of RL, the construction of the reward function assumes paramount importance [5, 6]. It defines the agent's objective and profoundly influences its learning process. A well-designed reward function serves as the guiding light for the agent, offering a clear signal about the desirability of different states and actions. Striking the right balance in the reward function is crucial, as it not only directs the agent toward its desired goals but also helps it steer clear of potential pitfalls. In essence, the reward function acts as a compass, shaping the agent's behavior and ultimately determining the success of the reinforcement learning process.

Supplementary Section 4: Benchmark PIC-RL on the scalable cliff walking task

A. Hyperparameters setting in PIC-RL model

The hyperparameters in the RL model will determine the performance of model training. In this section, we mainly analyze the influence of similarity coefficient β. It is noteworthy that when setting the target reward r_T to a small positive value, the single-step reward r must be negative to ensure the effectiveness of training. Otherwise, the agent may wander randomly and fail to locate the target point. On the one hand, when $\beta = 0$, the SRF degenerates to the constant reward function (CRF). On the other hand, β needs to be less than 1 to ensure the effectiveness of the training. Therefore, the similarity coefficient falls within the range of $[0, 1)$. We perform benchmark tests on the 10×10 cliff walking task using various similarity coefficients, and the training curves are illustrated in Fig. S6. The introduced similarity function guides the agent toward the target point, leading to a noticeable acceleration effect as β increases. When $\beta = 0.9$, the model training convergence speed of SRF on the 10×10 problem improves by 34.89% compared to CRF.

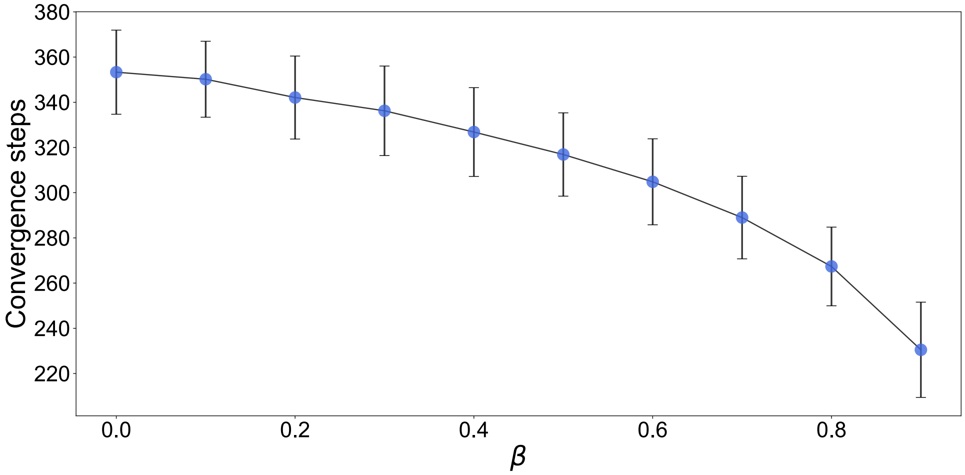

Fig. S6: **Results in the** 10×10 **cliff walking grid world for different similarity coefficient** β**.** Error bars denote one standard deviation taken over 500 agents.

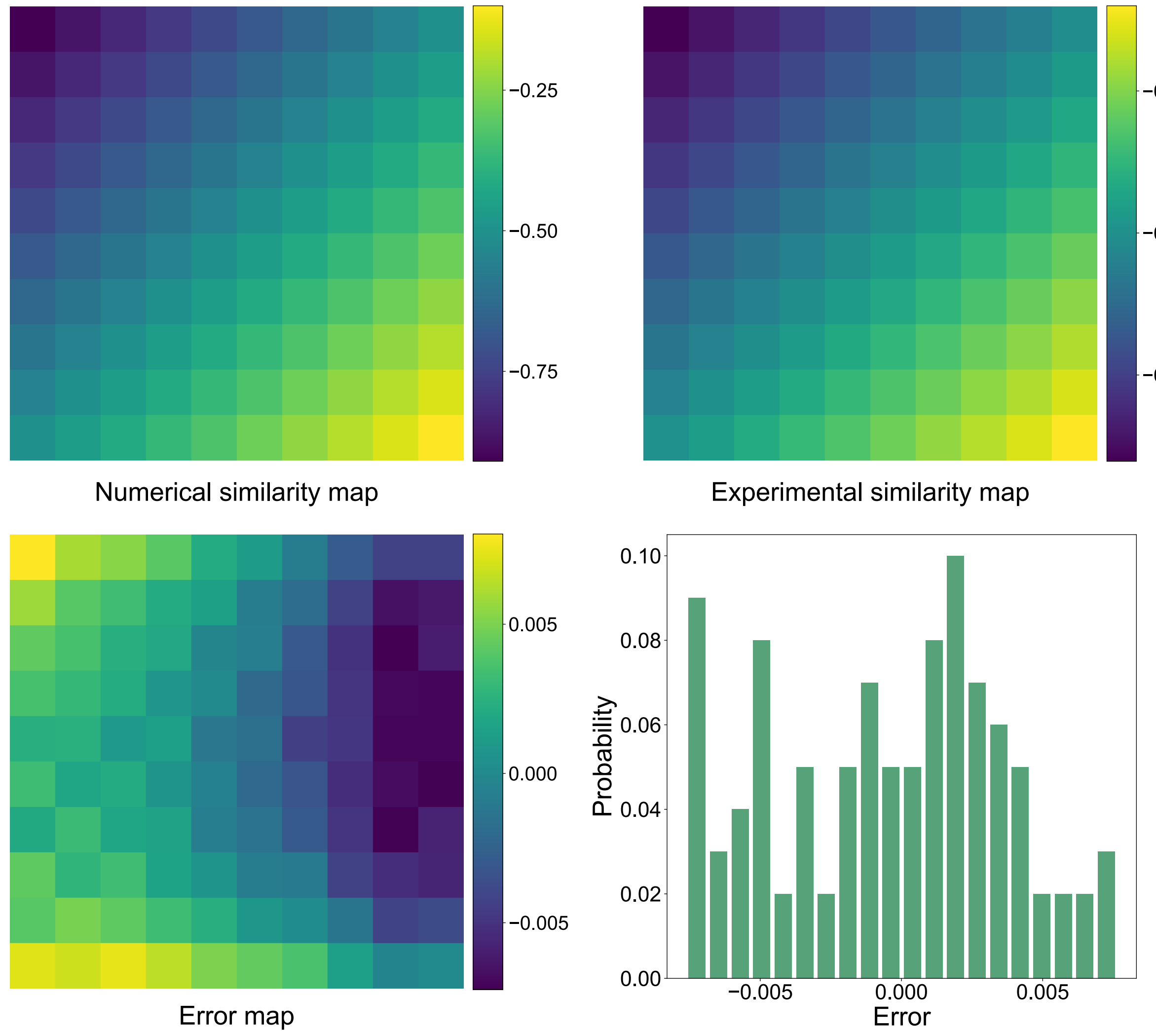

Fig. S7: **Similarity experimental data in** 10×10 **grid world environment.** Numerical **(a)** and experimental **(b)** results for the 10×10 grid world similarity map. **(c)** Error between experimental and numerical calculations, and **(d)** histograms illustrating the error distribution.

B. Scalability of SRF-based PIC-RL

In this scalability benchmark, we employ a square grid world and assess the convergence performance as a function of the square grid world dimension n, ranging from 10 to 100 in intervals of 10. The length of the cliff is $n-2$, situated at the bottom of the map and positioned between the starting point $(0, 0)$ and the target point $(0, n-1)$. There are no obstacles elsewhere on the map. We plot the curve illustrating the total number of steps growing with n and perform an exponential fit $y = ae^{bn} + c$ in Fig. S8. The fitting results reveal that the computational complexity of SRF is approximately $\mathcal{O}(n^2)$, while that of CRF is $\mathcal{O}(n^3)$. Therefore, in terms of the environment size n, SRF RL exhibits exponential training acceleration compared to CRF RL. Additionally, using advanced PIC to process large-scale similarity calculations with high computing power and low energy consumption will greatly improve the computing performance of the SRF algorithm.

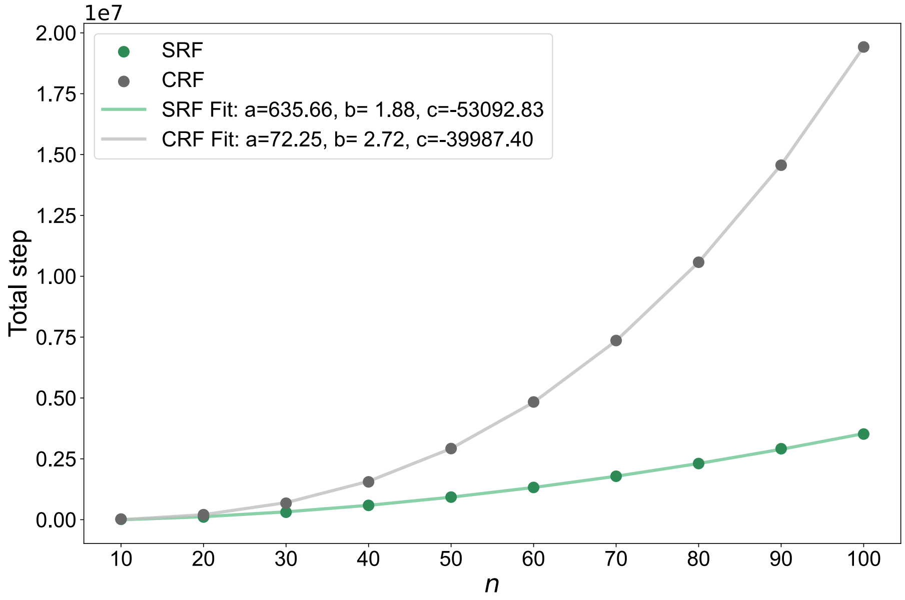

Fig. S8: **Scalability of SRF-based RL.** The total number of steps in the training process varies with the grid world size n for the cliff walking task under the SRF RL (green) and CRF RL (gray) algorithms. The line plots represent the results of exponential fitting to the data.

Supplementary Section 5: Background of the perovskites materials synthesis task

Two-dimensional Ruddlesden–Popper (RP) layered perovskites, known for their advantageous optoelectronic properties and excellent environmental stability, have attracted broad interest as potential lead-free high-performance photovoltaic absorbers [7]. The general chemical formula of 2D RP phase structure is $\mathrm{A}'_2[\mathrm{A}_{n-1}\mathrm{B}_n\mathrm{X}_{3n-1}]$, where $\mathrm{A}', \mathrm{A},$ and B denote different metal cations with stable valence states, balanced by the anion X. Du et al. [8] discovered a novel and stable 2D RP-type layered chalcogenide perovskite semiconductor $\mathrm{Ca_3Sn_2S_7}$ (CSS) based on first-principles GW calculations, which exhibits excellent optoelectronic characteristics. Hu et al. [9] designed the $\mathrm{Ca_6Sn_4S_{14-x}O_x}$ $(\mathrm{CSSO}_x, x\text{=}1\text{-}5)$ structure to increase the direct bandgap, rendering CSSO_x a candidate for new-generation photovoltaic absorbers.

Given the spatial structural ordering of RP-type CSSO_x perovskites, there are 3472 different possible derivative structures, constituting a vast structural space. Based on the density functional theory (DFT) results of total energy for all 3472 derivative structures related to each arrangement of mixed-atoms, structures labeled as $\mathrm{CSSO_1}$-11, $\mathrm{CSSO_2}$-85, $\mathrm{CSSO_3}$-072, $\mathrm{CSSO_4}$-0980, and $\mathrm{CSSO_5}$-1997 are identified as the most stable structures in different S : O ratios. Furthermore, the projected density of state (PDOS) and theoretical power conversion efficiency (PCE) for all five screened structures are calculated by DFT/PBE (Perdew–Burke–Ernzerhof) methods and Spectroscopic Limited Maximum Efficiency (SLME) method [10] respectively. The calculation results are summarized in Table S1. Based on the calculation results, $\mathrm{CSSO_4}$ exhibits the highest PCE. Therefore, we selected $\mathrm{CSSO_4}$-0980 as the privileged structure, serving as the target state in the RL algorithm. The performance of both $\mathrm{CSSO_2}$ and $\mathrm{CSSO_3}$ is inefficient, with the greater number of $\mathrm{CSSO_3}$ derivative structures contributing to a larger share of $\mathrm{CSSO_3}$ in the entire CSSO system. Therefore, we select $\mathrm{CSSO_3}$-072 as the starting state and investigate the synthesis path from $\mathrm{CSSO_3}$-072 to $\mathrm{CSSO_4}$-0980 to explore the

most effective method for improving the overall performance.

Table S1: The number of derivative structures and performance of CSSO_x.

Materials	Number of derivative structures	Bandgap[eV]	SLME
CSSO_1	14	0.70	22.3%
CSSO_2	91	0.12	4.58%
CSSO_3	364	0.23	5.49%
CSSO_4	1001	1.19	32.53%
CSSO_5	2002	1.02	29.72%

3472 different derivative CSSO_x structures are distinguished by the 14-dimensional atom vectors $\mathbf{V}(\mathbf{d})$, which consist of the distances between each atom and the central point in the crystal structure, arranged in order. The sequence and rules of atomic distances are shown in Fig. S9. In the atom vector $\mathbf{V}(\mathbf{d})$, the first $14-x$ bits denote the positions of the unsubstituted S atoms, while the last x bits represent the positions of the x O atoms. This encoding method can generate 3472 different vectors, which are utilized for similarity calculations of various material structures and SRF-RL training. We employ t-distributed stochastic neighbor embedding (t-SNE) [11] provided in the Python package scikit-learn [12] to visualize the high-dimensional state space. In addition to the location information, we calculate the electronegativity of the atoms and the total energy of the structure to characterize each structure more specifically. Finally, each material structure is encoded as a 29×1 vector $\mathbf{V}(\mathbf{d}, \mathbf{e}, E_{\mathrm{tot}})$ for the execution of the t-SNE model, where 28 bits for atomic distance-electronegativity pairs and the last one bit for the total energy E_{tot}. The initialization of t-SNE is conducted through principal components analysis (PCA), and the t-SNE model is used to discern the structural differences, yielding a 2×1 vector. The output of t-SNE allows for the visualization of the similarity between the 3472 structures of CSSO_x.

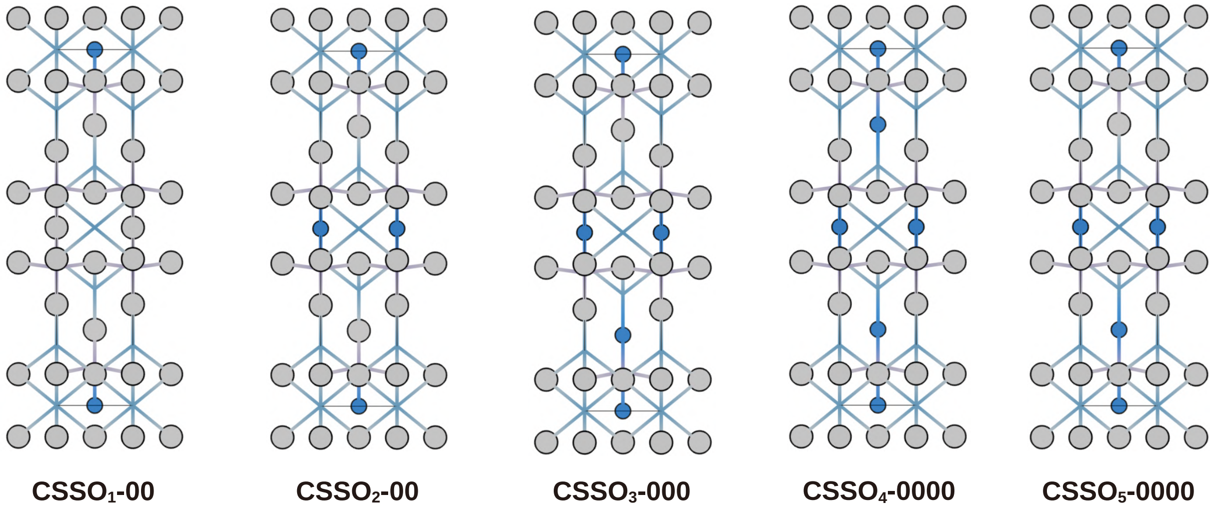

CSSO1-00	0.500	0.213	0.787	0.284	0.716	0.089	0.911	0.088	0.912	0.402	0.598	0.411	0.589	0.000
CSSO2-00	0.213	0.787	0.284	0.716	0.089	0.911	0.088	0.912	0.402	0.598	0.411	0.589	0.000	0.500
CSSO3-000	0.787	0.284	0.716	0.089	0.911	0.088	0.912	0.402	0.598	0.411	0.589	0.000	0.500	0.213
CSSO4-0000	0.284	0.716	0.089	0.911	0.088	0.912	0.402	0.598	0.411	0.589	0.000	0.500	0.213	0.787
CSSO5-0000	0.716	0.089	0.911	0.088	0.912	0.402	0.598	0.411	0.589	0.000	0.500	0.213	0.787	0.284

Fig. S9: **RP type CSSO$_x$ structure encoding method.** 2D schematic structures of RP type CSSO$_x$ and their corresponding $\mathbf{V}(\mathbf{d})$. Gray circles indicate S atoms, and blue circles indicate O atoms.

Supplementary Section 6: Comparison of different RL algorithms in perovskite materials synthesis task

In the main text, we show the application of Q-learning in solving complex perovskite material synthesis task. The pseudocode of PIC-RL Q-learning used in the main text is provided in Algorithm 1. Q-learning is one of the mainstream algorithms in RL, and other RL algorithms also have made great breakthroughs in recent years, such as deep-Q network (DQN), asynchronous advantage actor-critic (A3C), proximal policy optimization (PPO) and etc. Considering the complexity of the model and the discretization characteristics of the material synthesis problem, we mainly compare the learning efficiency of two classic RL algorithms: SARSA (state-action-reward-state-action) and Q-learning. SARSA is an on-policy RL algorithm, with the agent interacting with the environment and updating the policy based on actions taken. SARSA also uses Q-table to store action-value functions, and the decision-making part is the same as Q-Learning, and also uses the ϵ-greedy strategy. The difference is that the update methods of the two RL algorithms are different. We provide the complete pseudocode of SARSA in Alg. 2 for comparison with Q-learning.

Algorithm 1 PIC-RL Q-learning

```
Algorithm parameters: step size α ∈ (0, 1], small ϵ > 0
Initialize Q(s, a) = 0, for all ∀s in S†, a ∈ 𝒜(s)
Set Q(terminal, ) = 0
repeat
    Initialize S
    repeat
        Choose A from S using policy derived from Q (e.g., ϵ-greedy)
        Take action A and encode A into PIC, go to S′, observe R
        Q(S, A) ← Q(S, A) + α (r + γ · max_a Q(S′, a) − Q(S, A))
        S ← S′
    until S is terminal
until convergence
```

Algorithm 2 SARSA

```
Algorithm parameters: step size α ∈ (0, 1], small ϵ > 0
Initialize Q(s, a) = 0, for all ∀s in S†, a ∈ 𝒜(s)
Set Q(terminal, ) = 0
repeat
    Initialize S
    Choose A from S using policy derived from Q (e.g., ϵ-greedy)
    repeat
        Take action A, go to S′, observe R
        Choose A′ from S′ using policy derived from Q (e.g., ϵ-greedy)
        Q(S, A) ← Q(S, A) + α [R + γQ(S′, a′) − Q(S, A)]
        S ← S′ , A ← A′
    until S is terminal
until convergence
```

Comparing the algorithm processes of Q-learning and SARSA, it can be found that the main difference between the two algorithm lies in whether the behavior policy $\mu(a|s)$ and the evaluation(target) policy $\pi(a|s)$ are the same policy. The characteristic of on-policy learning (such as SARSA) is that the $\mu(a|s)$ and $\pi(a|s)$ are the same policy, while off-policy learning (such as Q-learning) generates the strategy $\mu(a|s)$ and the evaluation strategy $\pi(a|s)$ are different policies, specifically, the agent actually interacts with the environment through policy $\mu(a|s)$ generating actions, but uses the target policy $\pi(a|s)$ when updating the value of this state-action pair. Since off-policy learning is based on the results of past target policies, it has higher training efficiency in the complex high-dimensional state space of material synthesis than SARSA. Fig. S10 demonstrates the comparison of the learning curves of Q-learning and SARSA in the perovskite material synthesis task, both based on 100 agents and using the same similarity coefficient ($\beta = 0.5$). The learning efficiency of Q-learning increases by 46.15% compared to that of SARSA, which proves that Q-learning is more suitable RL algorithm for learning such tasks.

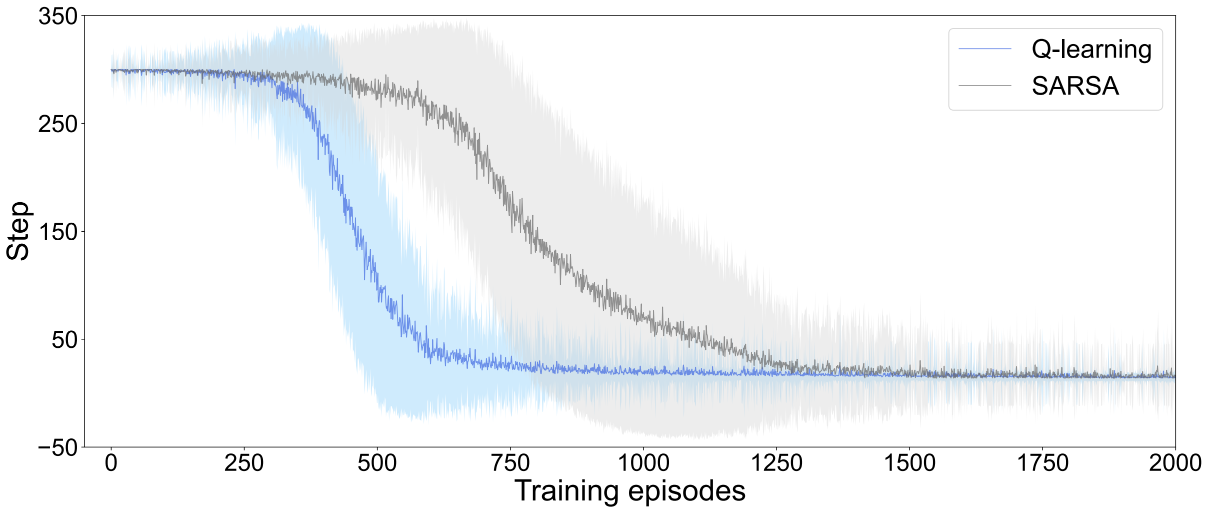

Fig. S10: **Comparison of training curves between Q-learning and SARSA in perovskite material synthesis tasks.**

Supplementary Section 7: Finite precision analysis of HyArch PIC and its impact on RL model

Device errors, such as splitter imbalance and phase encoding errors resulting from imperfect fabrication of the PIC, can compromise the numerical accuracy of the entire optoelectronic computing system. This, in turn, limits the scalability of the entire system and hinders its practicality and commercialization. We conduct finite precision analysis for the HyArch PIC, assessing its impact on PIC-RL model training.

We choose cosine distance $\mathcal{D}$ to quantify the numerical error of optical dot production operation:

$$\mathcal{D}(\mathbf{y}, \tilde{\mathbf{y}}) \equiv 1 - \frac{\mathbf{y} \cdot \tilde{\mathbf{y}}}{\|\mathbf{y}\| \|\tilde{\mathbf{y}}\|} \sim \frac{\sigma_y^2}{\overline{y_i^2}} \tag{S5}$$

where $\mathbf{y}$ is the ideal output vector and $\tilde{\mathbf{y}}$ is the output vector subject to noise. The approximate relationship can be obtained by introducing the error model expansion $\tilde{y}_i = y_i + \delta y_i$ into the definition, and $\sigma_y^2 = \mathrm{Var}\,(\delta y_i) = \mathrm{Var}\,(\tilde{y}_i)$ is the variance of δy_i.

As the HyArch PIC is a hybrid architecture consisting of unitary MZI mesh U and OCTOPUS module, the total cosine distance of HyArch PIC can be represented as:

$$\mathcal{D}_{\mathrm{H(N,M)}} = \mathcal{D}_{\mathrm{U(N/M)}} + \frac{N}{M}\mathcal{D}_{\mathrm{O(M)}} \tag{S6}$$

where $\mathrm{H}, \mathrm{U}, \mathrm{O}$ are abbreviations of HyArch PIC, unitary mesh and OCTOPUS respectively. To perform an $N \times N$ matrix-vector multiplication (MVM), we need an m-dimensional unitary MZI mesh $U(m)$ and m M-dimensional OCTOPUS modules ($M = N/m$) to form HyArch PIC, which is demonstrated in Fig. S11(a). We will analyze the cosine distance caused by U and OCTOPUS separately in the following.

The error model for the N-dimensional unitary matrix U is given in the [13] and can be expressed in the language of cosine distance as:

$$\mathcal{D}_{\mathrm{U(N)}} \sim \frac{N(N-1)}{2}\left(\epsilon^2\left(k_1\right) + \epsilon^2\left(k_2\right)\right) = 2(N-1)\sigma_{\mathrm{BS}}^2 \tag{S7}$$

The error model of OCTOPUS is deduced and analyzed in [14], but it should be noted that the OCTOPUS in this paper is a modified version of the theoretical scheme. We add an additional beam splitter tree and a column of MZI for the encoding of the input light, so they also need to be taken into account when analyzing the error model. By assuming that the variance of the interferometer and attenuator is equal to twice the BS variance $\sigma_I^2 = \sigma_{\mathrm{A}}^2 = 2\sigma_{\mathrm{BS}}^2$, we can derive the cosine distance of the modified N-dimensional OCTOPUS model in a similar way:

$$\mathcal{D}_{\mathrm{O(N)}} \sim 4(\log(N) + 1)\sigma_{\mathrm{BS}}^2 \tag{S8}$$

The cosine distance of the optical dot product by HyArch PIC can be represented as:

$$\mathcal{D}_{\mathrm{H(N,M)}} = \frac{4N}{M}\left(\log(M) + 1 - \frac{M}{2N}\right)\sigma_{\mathrm{BS}}^2 \tag{S9}$$

and when N is large, we assume that $N = M^2$, so we have

$$\mathcal{D}_{H(N)} \sim 2\sqrt{N}\log(N)\sigma_{\mathrm{BS}}^2 \tag{S10}$$

The singular-value-decomposition (SVD) based network is the dominant architecture for implementing MVM operation, and the matrix is decomposed as $\mathbf{W} = \mathbf{V}^{\dagger}\mathbf{\Sigma}\mathbf{U}$, where $\mathbf{U}$ and $\mathbf{V}^{\dagger}$ can be implemented using an N-dimensional unitary MZI mesh, and $\mathbf{\Sigma}$ can be implemented using a column of MZI array. Therefore, $2N + 1$ layers are needed for performing optical SVD, and the cosine distance of SVD architecture is $4N\sigma_{\mathrm{BS}}^2$. To summarize, the error scale of the HyArch PIC is $\mathcal{O}(\sqrt{N}\log(N))$, providing a sub-exponential advantage over the SVD architecture PIC with $\mathcal{O}(N)$. We assess the robustness of various architectures in conducting an N-dimensional arbitrary real-value MVM operation, as depicted in Figure S11(b). Our evaluations consider different values of N, ranging from $N = \{4, 8, 16, 32, 64, 128, 256\}$, and corresponding dimensions for the vector M, denoted as $M = \{1, 2, 4, 4, 8, 16, 16\}$. Specifically, as N approaches 64, we observe that the overall error levels are approximately equal for the HyArch PIC configuration with $\sigma_{\mathrm{BS}} = 0.005$ and the SVD PIC configuration with $\sigma_{\mathrm{BS}} = 0.01$.

In summary, we present a comparative analysis in Table S2 of integrated optical networks designed for N-dimensional optical dot product operations. Our analysis highlights the significant advantages of the HyArch PIC architecture over MZI mesh structures, including U network and SVD network, in terms of both computational accuracy and integration capabilities when conducting optical dot product operations. Given that high-dimensional dot product operations serve as the cornerstone of PIC-RL, the scalability and precision of the HyArch PIC architecture are paramount to the success of PIC-RL applications.

Table S2: Comparative analysis of integrated optical network architectures for optical dot product operations

	Error scaling	Network depth	Number of modulators
U network	$2N\sigma_{\mathrm{BS}}^2$	N	$\sim N^2$
SVD network	$4N\sigma_{\mathrm{BS}}^2$	$2N+1$	$\sim 2N^2$
HyArch PIC	$2\sqrt{N}\log(N)\sigma_{\mathrm{BS}}^2$	$\sqrt{N}+2$	$\sim 4N$

We further investigate the effect of finite numerical precision on the RL training convergence speed, as shown in Fig. S11(c). Using a 10×10 size cliff walking task to simulate the training convergence speed of 500 SRF agents under $\beta = 0.5$ with different cosine distances, we find that if the cosine distance is kept within 10%, it has little effect on the training of the low-dimensional RL model. The training convergence speed of the model decreases significantly with the increase in cosine distance. As N and β increase, minimizing the calculation error of similarity becomes increasingly crucial. In a large-scale search space, excessive errors can impede the model from converging successfully. Hence, the ongoing enhancement of fabrication processes to minimize device errors and the exploration of advanced calibration methods for mitigating system errors will be pivotal focal points in the advancement of future photonic technologies [15–17].

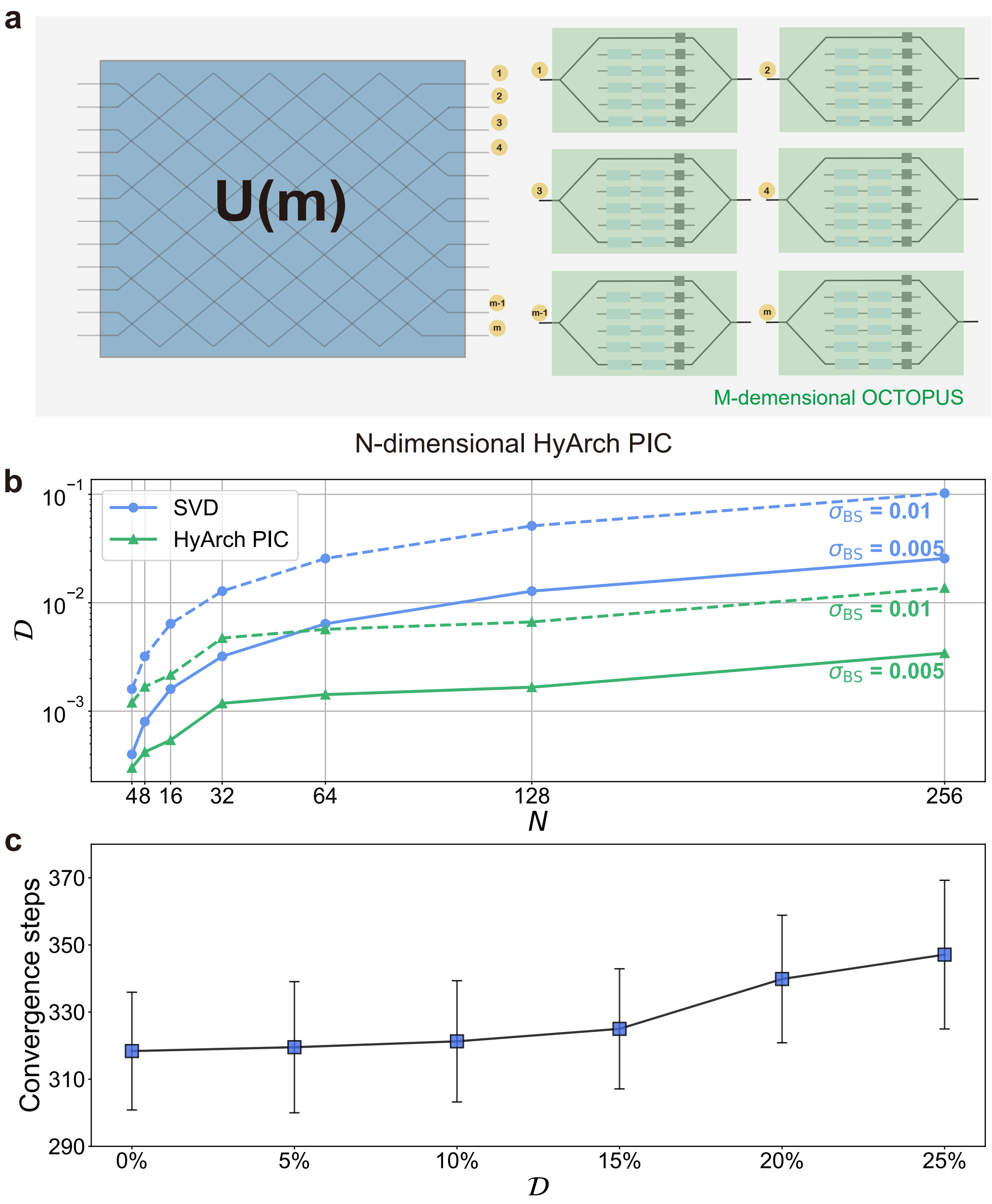

Fig. S11: **Precision analyses and scaling performance of HyArch PIC. (a)** Schematic diagram of the scalable HyArch PIC with m input ports and m output ports. The m output ports of the MZI mesh $U(m)$ are connected to m M-dimensional OCTOPUS modules. **(b)** Scaling of the cosine distance $\mathcal{D}$ with mesh size N, illustrating the qualitative scaling difference between the SVD structure PIC and the HyArch PIC. **(c)** Convergence steps as a function of cosine distance $\mathcal{D}$. The benchmark RL environment is a 10×10 grid world cliff walking task, and each data point is calculated by 500 agents. Error bars denote one standard deviation taken over 500 agents.

Supplementary Section 8: Computing power and energy efficiency analysis

The development of advanced photonic technologies has made optoelectronic hybrid computing architecture one of the most promising candidates for next-generation AI hardware platforms. In this section, we construct a model to analyze the potential advantages of the HyArch PIC architecture in terms of computing power and energy consumption.

To demonstrate the advantages of photonic technology, we assume our HyArch PIC integrates the high-speed electro-optic modulators (EOM) [18] and micro-electromechanical systems (MEMS) phase modulation unit [19,20]. EOMs are used to encode different states to compute similarity with MEMS-encoded target state, and the phases of all the tail phase shifters are stabilized by MEMS cells. We explore the computing power of photonic computing at various system dimensions N, wherein the system dimension refers to the hyper-HyArch PIC with N EOMs, highlighting its remarkable scalability. M-dimension dot-product needs $2M-1$ floating point operations in digital electric hardware, while in the M-dimensional OCTOPUS module, the input optical signal can be transmitted through the encoded network to streamline the M-dimensional dot product operation into a single operation. Therefore, the number of operations per second (OPS) R of HyArch PIC can be expressed as:

$$R = \frac{N}{m}(2M-1)f_{\mathrm{s}}\ \mathrm{OPS} \sim M(2M-1)f_{\mathrm{s}}\ \mathrm{OPS} \tag{S11}$$

where f_{s} is the system clock frequency. The approximation condition assumes that at large total dimension N the OCTOPUS dimension M will be approximated by $\sqrt{N}$.

It should be noted that the bandwidth of a single EOM modulator can exceed 100 GHz [18], but the system frequency of the EOM-based PIC network may be constrained by the multi-channel electrical driver. In our comprehensive feasibility assessment [21–25], we conducted calculations to evaluate the computing power of the hyper-HyArch PIC system at three distinct

operating frequencies: 100 MHz, 1 GHz, and 10 GHz. These results were then compared to the computing power of corresponding electrical hardware and artificial intelligence models. Our HyArch PIC architecture exhibits high scalability, allowing for significant expansion by increasing the size of the monolithic network [25] and employing inter-chip optical interconnect technology [26,27]. Fig. S12 illustrates the relationship between computing power and network size (M), revealing that the computing power of HyArch PIC grows quadratically with M. As depicted in Fig. S12, even with a 100 MHz electric drive system, the computing power offered by a relatively small-scale HyArch PIC system is sufficient to meet the computational demands of practical neural networks like AlexNet and ResNet-152 [28]. Furthermore, when operating in synergy with 10 GHz drivers, 128-dimensional HyArch PIC systems can provide computing power comparable to that of the A100, which serves as a primary source of computational capability in modern data centers.

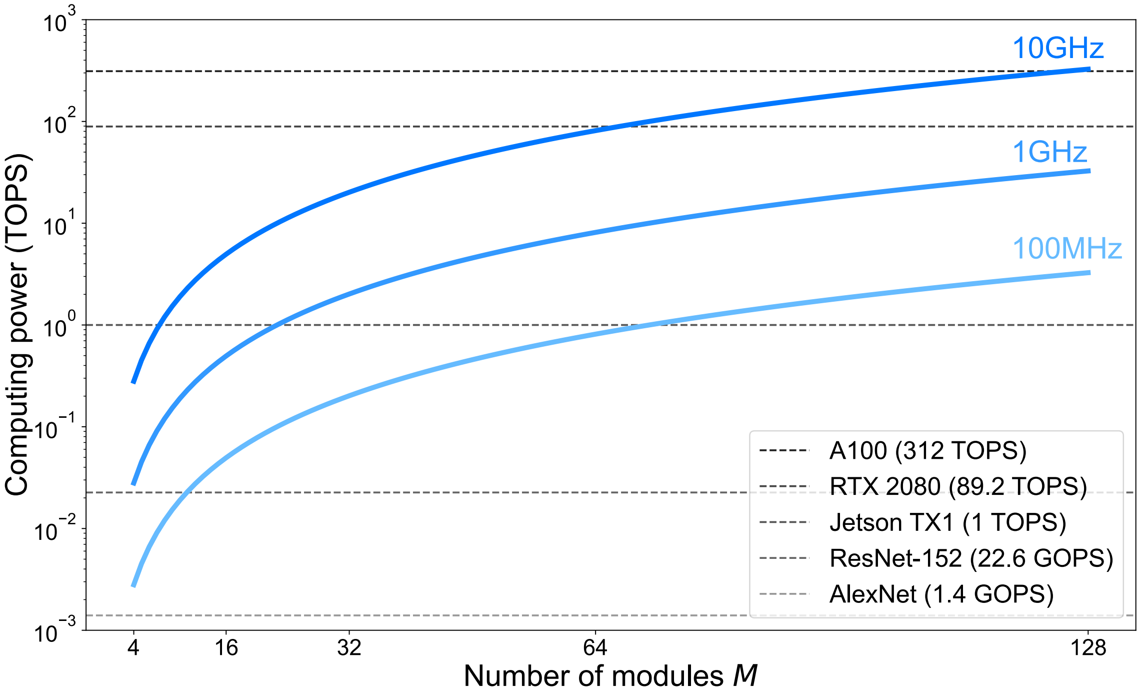

Fig. S12: **Computing power of hyper-HyArch PIC as a function of module number** M **at different system frequencies.**

In general, the energy consumption of HyArch PIC system can be divided into four major parts: (1) optical power loss from laser to detector P_{opt}; (2) the transmitter port power consumption required to drive the EOM array and digital-to-analog conversion (DAC) $P_{\mathrm{T}} = P_{\mathrm{EOM}} + P_{\mathrm{DAC}}$; (3) the receiver power consumption required by the transimpedance amplifier (TIA) and ADC to achieve amplification and analog-to-digital conversion of the output optical signal $P_{\mathrm{R}} = P_{\mathrm{TIA}} + P_{\mathrm{ADC}}$; and (4) the power consumption required by MEMS weight array and mesh P_{MEMS}. In $N = M^2$ dimension HyArch PIC, there are M input/output ports each and M^2 EOMs, therefore M ADCs and M^2 DACs are needed. The total computation efficiency can be expressed as:

$$\eta_{\mathrm{total}} = \frac{P_{\mathrm{opt}} + M^2(P_{\mathrm{EOM}} + P_{\mathrm{DAC}}) + M(P_{\mathrm{ADC}} + P_{\mathrm{TIA}}) + (M(M-1)/2 + 2M^2)P_{\mathrm{MEMS}}}{M(2M-1)f_{\mathrm{s}}} \tag{S12}$$

In the advanced PIC process, the power consumption of the optical components is nearly negligible when compared to the high-energy demands of the high-speed electrical drive. Below we mainly calculate the power consumption of the electrical part. We simulated the lithium niobate on insulator (LNOI) EOM model through finite element analysis and calculated that the equivalent capacitance of 5 mm LNOI EOM is 0.3 pF to estimate the power consumption of EOM. Assuming a 2 V drive voltage at 1 GHz, the power consumption of EOM is $P_{\mathrm{EOM}} = CV^2\Delta f_{\mathrm{EOM}} = 1.2$ mW . At 1 GHz system frequency, the power consumption of DAC/ADC/TIA is 11.06 mW, 2.55 mW and 57 mW respectively. When M is much greater than 1, the above formula can be simplified as:

$$\eta_{\mathrm{total}} \sim \frac{1}{2f_{\mathrm{s}}}\left(P_{\mathrm{T}} + \frac{P_{\mathrm{R}}}{M} + 2.5P_{\mathrm{MEMS}}\right) \tag{S13}$$

Theoretical energy efficiency calculation result for a 128-dimensional HyArch PIC operating at 1 GHz is 5.86 pJ/OP, and most of the energy consumption comes from the high-speed

DAC (94.45%). Further reducing the power consumption of the DAC can bring the energy efficiency of the whole system close to the commercial GPU.

Table S3: Summary of parameters in numerical calculations

Category		value	ref
Capacity of EOM		0.3 pF	Simulated
DAC (transmitter, 1 GHz)		11.06 mW	[29]
ADC (receiver, 1 GHz)		2.55 mW	[30]
TIA (receiver, 1 GHz)		57 mW	[31]
MEMS (weight, stable)		75 μW	[19]
Computing power	JETSON TX1	1 TOPS	[32]
	RTX 2080	89.2 TOPS	[33]
	A100	312 TOPS	[34]
Power consumption	JETSON TX1	15 W	[32]
	RTX 2080	225 W	[33]
	A100	400 W	[34]

Supplementary Section 9: Fully integrated optoelectrical RL computing scheme

Based on the existing PIC fabrication processing, most integrated ONN demonstrations are realized by linear optical networks to implement the fully connected layer and convolutional layer operations in the neural network, which limits the further combination of PIC and advanced AI algorithms. It is gratifying to see that there is increasing research being conducted on nonlinear integrated cells and optoelectronic system on a chip (SoC), which will greatly enhance the versatility of optoelectronic computing. The hybrid integration of photonic circuits and electronic circuits to realize optoelectronic SoC is the trend of future computing hardware development.

Specifically, in reinforcement learning, the reward function plays the role of ”evaluating” the agent’s behavior, and its mathematical expression will determine the final performance of the model. We introduce cosine similarity in the reward function and validate its effectiveness for RL training in finite discrete environment space, while in the infinite or continuous environment

space, this method needs to be improved. The Q-learning method of using tables to store action values is only applicable when the state and actions of the environment are discrete and the space is relatively small. When the number of states or actions is very large or continuous, there are infinite state-action pairs, and we cannot use Q-table to record all Q values of each state-action pair. Therefore, when the problem is oversize or continuous, we need to use function fitting to estimate values, that is, treat this large-scale value table as data and fit it by a parameterized function. Due to the powerful expressive ability of neural networks, we can use a neural network to represent the Q function and the neural network is called deep Q network (DQN). The loss function of DQN can be expressed as:

$$L(\mathbf{w})=\mathbb{E}\left[\left(r+\gamma \max_{a'} Q\left(s', a', \mathbf{w}^{-}\right)-Q(s, a, \mathbf{w})\right)^{2}\right] \tag{S14}$$

where the $\mathbf{w}$ is the weight of Q-network. Linear optical networks can replace electronic computing hardware for implementing linear neural networks to train Q functions in DQN. On-chip electro-optic conversion and optic-electric conversion could be realized by EOM and Ge photodetector, respectively. Real-time iterations and updates are possible with the help of ultra-low-loss optical delay lines. The acquisition of the Q function can be realized by simulating the interaction between the agent and the environment through HyArch PIC, and the update and storage of the Q function and the nonlinear part of the algorithm can be assigned to the on-chip electronic unit. The fully integrated optoelectronic DQN scheme is shown in Fig. S13.

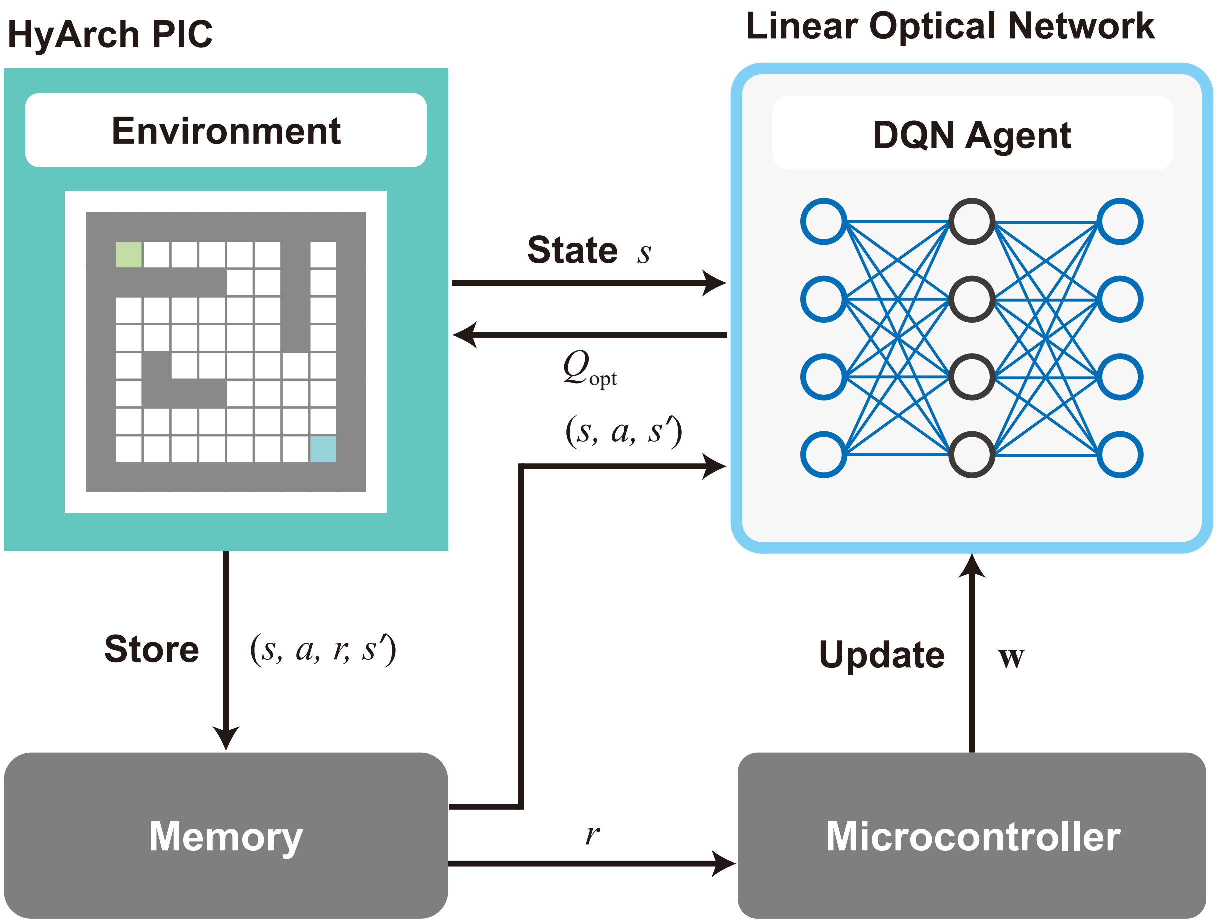

Fig. S13: **Schematic of the fully integrated optoelectrical DQN architecture.** A multilayer photonic network functions as the deep neural network for the deep Q-learning network (DQN) agent. The HyArch PIC serves as the environment that interacts with the DQN agent. Following each interaction, rewards are transmitted to on-chip memory for iteration and Q-table updates. The microelectronic control unit provides the policy to the DQN agent for the subsequent episode of actions.

Supplementary Reference

[1] Scikit-optimize, `https://scikit-optimize.github.io/` (2023).

[2] Sutton, R. & Barto, A. *Reinforcement Learning: An Introduction* (MIT press, 2018).

[3] Watkins, C. J. & Dayan, P. Q-learning. *Mach. Learn.* **8**, 279–292 (1992).

[4] Mnih, V. *et al.* Human-level control through deep reinforcement learning. *Nature* **518**, 529–533 (2015).

[5] Van Seijen, H. *et al.* Hybrid reward architecture for reinforcement learning. *Adv. Neural Inf. Process. Syst.* **30** (2017).

[6] Icarte, R. T., Klassen, T. Q., Valenzano, R. & McIlraith, S. A. Reward machines: Exploiting reward function structure in reinforcement learning. *J. Artif. Intell. Res.* **73**, 173–208 (2022).

[7] Tsai, Hsinhan, *et al*. High-efficiency two-dimensional Ruddlesden–Popper perovskite solar cells, *Nature* **536**, 312-316 (2016).

[8] Du, J., Shi, J. J. 2D $\mathrm{Ca_3Sn_2S_7}$ chalcogenide perovskite: a Graphene-like semiconductor with direct Bandgap 0.5 eV and ultrahigh carrier mobility $6.7 \times 10^4 cm^2 V^{-1} s^{-1}$, *Adv. Mater.* **31**, 1905643 (2019).

[9] Hu, J., *et al.*. Accelerated design of photovoltaic Ruddlesden–Popper perovskite $\mathrm{Ca_6Sn_4S_{14-x}O_x}$ using machine learning. *APL Mater.* **8**, 111109 (2020).

[10] Yu, L., Zunger, A. Identification of potential photovoltaic absorbers based on first-principles spectroscopic screening of materials. *Phys. Rev. Lett.* **108**, 068701 (2012).

[11] Van Der Maaten, *et al.* Accelerating t-SNE using tree-based algorithms. *J. Mach. Learn. Res.* **15**, 3221-3245 (2014).

[12] Scikit-learn, `https://scikit-learn.org/` (2023).

[13] Bandyopadhyay, *et al.* Hardware error correction for programmable photonics. *Optica* **8**, 1247-1255 (2021).

[14] Zhang, X. M., Yung, M. H., Low-depth optical neural networks. *Chip* **1**, 100002 (2022).

[15] Song, Lijia, *et al.* Mach–Zehnder silicon-photonic switch with low random phase errors. *Opt. Lett.* **46**, 78-81 (2021).

[16] Papadovasilakis, *et al.* Fabrication tolerant and wavelength independent arbitrary power splitters on a monolithic silicon photonics platform. *Opt. Express* **30**, 33780-33791 (2022).

[17] Sun, Tianyu, and Mingjun Xia. Low loss modified Bezier bend waveguide. *Opt. Express* **30**, 10293-10305 (2022).

[18] Xu, M., *et al.* Dual-polarization thin-film lithium niobate in-phase quadrature modulators for terabit-per-second transmission. *Optica* **9**, 61-62 (2022).

[19] Gyger, S., *et al.* Reconfigurable photonics with on-chip single-photon detectors. *Nat. Commun* **12**, 1408 (2021).

[20] Quack, N.,*et al.* Integrated silicon photonic MEMS. *Microsyst. Nanoeng* **9(1)**, 27 (2023).

[21] Ying, Z. *et al.* Electronic-photonic arithmetic logic unit for high-speed computing. *Nat. Commun.* **11**, 2154 (2020).

[22] Dong, M. *et al.* High-speed programmable photonic circuits in a cryogenically compatible, visible–near-infrared 200 mm CMOS architecture. *Nat. Photonics* **16**, 59–65 (2022).

[23] Mourgias-Alexandris, G. *et al.* Noise-resilient and high-speed deep learning with coherent silicon photonics. *Nat. Commun.* **13**, 5572 (2022).

[24] Lin, Z. *et al.* High-performance polarization management devices based on thin-film lithium niobate. *Light: Sci. Appl.* **11**, 93 (2022).

[25] Peng, B., Hua, S., Su, Z., Xu, Y. & Shen, Y. A 64× 64 integrated photonic accelerator. In *2022 IEEE Photonics Conference (IPC)*, 1–2 (IEEE, 2022).

[26] Abrams, N. C. *et al.* Silicon photonic 2.5 D multi-chip module transceiver for high-performance data centers. *J. Lightwave Technol.* **38**, 3346–3357 (2020).

[27] Hummingbird: Lightelligence optical network-on-chip accelerator. `https://www.lightelligence.ai/index.php/product/hummingbird.html`

[28] Zhang, M. *et al.* Optimized compression for implementing convolutional neural networks on fpga. *Electronics* **8**, 295 (2019).

[29] Demirkiran, C. *et al.* An electro-photonic system for accelerating deep neural networks. *ACM J. Emerg. Technol. Comput. Syst.* **19**, 1-31 (2023).

[30] Oh, D.-R. *et al.* An 8b 1GS/s 2.55 mW SAR-flash ADC with complementary dynamic amplifiers. In *2020 IEEE Symposium on VLSI Circuits*, 1–2 (IEEE, 2020).

[31] Sedighi, B. & Scheytt, J. C. Low-power SiGe BiCMOS transimpedance amplifier for 25-GBaud optical links. *IEEE Transactions on Circuits and Systems II: Express Briefs* **59**, 461–465 (2012).

[32] NVIDIA JETSON TX1 specifications. Available from: `https://images.nvidia.com/content/tegra/embedded-systems/pdf/JTX1-Module-Product-sheet.pdf`

[33] NVIDIA GeForce RTX 2080 specifications. Available from: https://images.nvidia.cn/aem-dam/Solutions/geforce/ada/nvidia-ada-gpu-architecture.pdf

[34] NVIDIA A100 PCIe 80 GB specifications. Available from: https://images.nvidia.cn/aem-dam/en-zz/Solutions/data-center/nvidia-ampere-architecture-whitepaper.pdf

[35] Bandyopadhyay, S. *et al.* Single chip photonic deep neural network with accelerated training. Preprint at *https://doi.org/10.48550/arXiv.2208.01623* (2022).
