# Supplementary material for: High-efficiency reinforcement learning with hybrid architecture photonic integrated circuit
Source: Nat Commun. 2024 Feb 5;15:1044. doi: 10.1038/s41467-024-45305-z (PMC10844654; doi:10.1038/s41467-024-45305-z)
Supplement: Supplementary file 3 — Description of Additional Supplementary Files [file 41467_2024_45305_MOESM3_ESM.docx]

**Supplementary Data Legend**

**File Name: Supplementary Data 1**

**Description:** This file, "Supplementary Data 1," contains atom vectors for all 3472 derivative CSSO_x structures obtained from <https://doi.org/10.1063/5.0022007>. The data is presented in a 3472x14 matrix stored in a .csv file, with each row recording the atom vectors corresponding to distinct derivative CSSO_x structures in sequential order. The encoding method is comprehensively explained in Supplementary Section 5. These data are utilized for the similarity calculation phase of the SRF in the perovskite materials synthesis task with PIC-RL.
